# Venom Ex Machina? Exploring the Potential of Cell-Free Protein Production for Venom Biodiscovery

**DOI:** 10.3390/ijms25158286

**Published:** 2024-07-29

**Authors:** Anne Paas, Josephine Dresler, Lea Talmann, Andreas Vilcinskas, Tim Lüddecke

**Affiliations:** 1 Branch for Bioresources, Fraunhofer Institute for Molecular Biology and Applied Ecology, Ohlebergsweg 12, 35392 Gießen, Germany; josephine.dresler@ime.fraunhofer.de (J.D.); andreas.vilcinskas@ime.fraunhofer.de (A.V.); 2 LOEWE-Centre for Translational Biodiversity Genomics (LOEWE-TBG), Senckenberganlage 25, 60325 Frankfurt, Germany; 3 Syngenta Crop Protection, Werk Stein, Schaffhauserstrasse, CH4332 Stein, Switzerland; lea.talmann@syngenta.com; 4 Institute for Insect Biotechnology, Justus Liebig University of Gießen, Heinrich-Buff Ring 26-32, 35392 Gießen, Germany

**Keywords:** venom biotechnology, bioprospection, toxinology, cytotoxin, phospholipase D

## Abstract

Venoms are a complex cocktail of potent biomolecules and are present in many animal lineages. Owed to their translational potential in biomedicine, agriculture and industrial applications, they have been targeted by several biodiscovery programs in the past. That said, many venomous animals are relatively small and deliver minuscule venom yields. Thus, the most commonly employed activity-guided biodiscovery pipeline cannot be applied effectively. Cell-free protein production may represent an attractive tool to produce selected venom components at high speed and without the creation of genetically modified organisms, promising rapid and highly efficient access to biomolecules for bioactivity studies. However, these methods have only sporadically been used in venom research and their potential remains to be established. Here, we explore the ability of a prokaryote-based cell-free system to produce a range of venom toxins of different types and from various source organisms. We show that only a very limited number of toxins could be expressed in small amounts. Paired with known problems to facilitate correct folding, our preliminary investigation underpins that venom-tailored cell-free systems probably need to be developed before this technology can be employed effectively in venom biodiscovery.

## 1. Introduction

Venom is a key evolutionary innovation that evolved convergently multiple times in a diverse range of organisms [1,2,3]. In animals, it serves mostly predation, defense and intraspecific competition [4]. These biological functions are facilitated by the bioactive components of a venom, most of which are proteins and peptides referred to as toxins. These were evolutionarily refined to target key physiological targets over millions of years of Darwinian evolution [2]. As a result, venom toxins belong to the most potent and most selective naturally occurring molecules known to us. While this grants them the ability to cause tremendous damage in scenarios of envenomation, the same destructive molecules are also candidates for further modification to be subsequently translated into drug leads [5,6,7].

In the past, venom biodiscovery for novel therapeutics has been primarily bioactivity-driven [8]. In such classic experiments, a venom is fractionated by chromatography and the yielded fractions are subsequently investigated for structural properties (e.g., via mass spectrometry, crystallography or NMR-spectroscopy) and/or function (via bioassays). This traditional workflow has proven itself to be a powerful approach towards venom biodiscovery and has already generated several drugs, including Captopril, Exenatide and Ziconotide [5]. However, it is, application-wise, restricted to taxa with rather high potential venom yields, as usually several milligrams of dried venom are required to employ it effectively [8,9,10]. That said, large swathes of venomous biodiversity are rather small invertebrates that do not deliver method-compatible amounts of venom, but their venoms are still valuable for biomedical applications [10,11,12,13,14]. In these cases, a large number of individuals are required to run even a single experiment and quite frequently, venom collection fails completely due to the small size of the venom apparatus. In order to incorporate such small and difficult to study venomous animals (e.g. pseudoscorpions, small spiders or insects) into future biodiscovery programs, novel technological answers are required.

One of the potential solutions to this persistent obstacle may lie in the application of biotechnology [15]. Cell-free production technologies are especially attractive in this regard [15,16]. In such cell-free systems, an artificial cell lysate is generated that contains all major components of the hosts’ genetic machinery required to perform protein biosynthesis [15]. By adding a recombinant piece of DNA encoding a protein in the hosts’ codon usage and under the control of a suitable promotor, these systems are theoretically able to synthetize any inserted venom toxin of interest. They are especially promising because protein synthesis is often facilitated in only a few hours. A range of cell-free systems are commercially available and optimized to carry out protein production with simple protocols, allowing parallelization, and thus enabling a high throughput [17,18,19,20]. Lastly, the generation of genetically modified organisms is circumvented. Overall, these cell-free systems, at first glance, promise to solve a range of persistent problems for venom biodiscovery.

However, so far, only very limited knowledge upon the suitability of cell-free production systems in light of venom biodiscovery is available. In the past, we have explored the promises and pitfalls of cell-free protein production for venom biodiscovery using a toxin from a sicariid spider as an example [21]. Thereby, we discovered that out of the tested systems, a prokaryote-based product appears to be the best suited system to produce venom toxins and, besides our preliminary work, a few other attempts were made to explore cell-free systems. Yet, those works usually only focused on a small diversity of toxins from only a few lineages (spiders, snakes and bees specifically) [22,23,24], while a comprehensive study using a broader assembly of venom toxins from different origins remains to be performed. Here, we provide a first exploration of the suitability of a prokaryote-based cell-free system on multiple toxins from various host organisms. We attempt to shed light on the ability of the chosen system to produce meaningful amounts of selected toxins from several animal lineages. Therewith, we aim to partially close this important research gap and to provide a first assessment of the general suitability of cell-free systems in light of venom biodiscovery and thus spark future, more detailed investigations.

## 2. Results

### 2.1. Gene Constructs for Cell-Free Production of Toxins

In order to explore the suitability of cell-free systems for venom biodiscovery, we selected a previously used prokaryote system (NEB PURExpress In Vitro Protein Synthesis System).

We selected a total of 30 toxins of medically and/or pharmacologically interest from snakes, arachnids, insects and leeches from entries in public databases and previous publications. As we opted to explore the functionality of the cell-free system, we selected a diverse range of venom components across a broader taxonomic scale and diverse protein families with different properties and molecular size. The chosen toxins belonged to diverse toxin groups with different targets, including neurotoxic peptides with an inhibitory cysteine knot motif (ICK), cytotoxic three finger toxins (3Ftx), and enzymes. The molecular sizes covered a range from only 3.1 kDa up to 45.3 kDa. A full overview of the selected toxins is given in Table 1 and full information of these toxins with sequences are provided in Supplementary Table S1. For expression, we created suitable gene constructs as outlined previously and employed the chosen cell-free system on them according to the manufacturer’s recommendations.

### 2.2. Limited Success Rates of Cell-Free Expression

After attempting cell-free expression of our selected toxins, we employed SDS-PAGE profiling to validate whether protein production has occurred as outlined previously. We compared the presence/absence of Coomassie-stained protein bands at expected sizes between the expression batches and the control lanes. The intensity of the bands serves as an approximation of the recovered protein yield. Our investigation revealed that only a marginal fraction of the selected proteins was seemingly produced. Of all the investigated toxins, only four, belonging to two distinct toxin types were successfully synthesized. The first two successfully synthesized toxins were cytotoxic 3Ftx from cobras (genus *Naja*). One was Sagitoxin (Uniprot ID = P83345) from the Andaman cobra, *Naja sagittifera*. The other was Cytotoxin 1 (Uniprot ID = P01455) from the snouted cobra, *Naja annulifera*. Both of these are relatively short members of the short-chain subfamily of 3Ftx and are composed of 60 amino acids and have a molecular weight of <7 kDa. Both contain four disulfide bonds. The other two successfully expressed components are toxic phospholipase D (PLD) enzymes of arachnid origin. One (Uniprot ID = A0A1L4BJ98) was previously isolated from the Gadim scorpion, *Hemiscorpius lepturus*. The other PLD was originally described from venom of the recluse spider, *Loxosceles laeta* (Uniprot ID = Q1KY80). Similar to the expressed 3Ftx, these PLDs are known for their ability to cause tissue damage. However, in contrast to the 3Ftx, the PLDs cause these effects through enzymatic cleavage of phosphodiester bonds between phospholipids, including sphingomyelin, that are present in cell membranes [26,27]. Both represent rather large molecules with 288 amino acids and a molecular weight of 33 kDa (*H. lepturus*) and 285 amino acids with a molecular weight of 32 kDa (*L. laeta*). As for modifications, they contain two (*H. lepturus*) and one (*L. laeta*) disulfide bonds, respectively.

**Figure 1:**
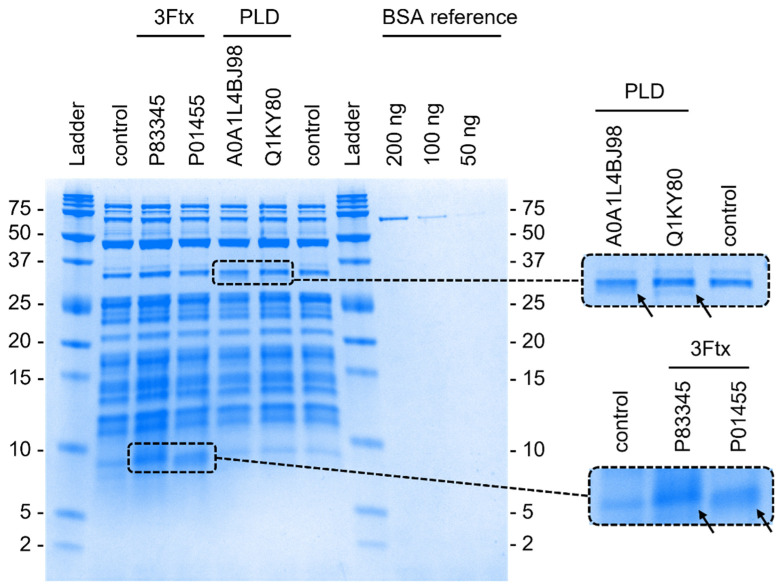
SDS-PAGE of successfully produced 3Ftxs (P83345, P01455) and PLDs (A0A1L4BJ98, Q1KY80), including a protein ladder and control and BSA references.

In most cases, the intensities of the bands were rather low, indicating that the yield of produced toxin was only minuscule. A comparison of the obtained results with our BSA reference led us to estimate that 1.5 µL of both cytotoxic 3Ftx contained approximately 200 ng of protein, resulting in a yield of <133 mg/L protein. In contrast, each of the two Phospholipase D contained approximately 100 ng of protein, yielding <67 mg/L expressed enzyme. Figure 1 presents an SDS-PAGE gel with the successfully produced toxins as well as our BSA reference, with the objective of estimating the yield of the expressed proteins. 

## 3. Discussion

Cell-free production has recently been suggested as a potentially powerful addition to the methodological repertoire in zootoxinology [15,16]. However, only a few studies have previously attempted to employ it for venom biodiscovery and those usually focused on selected toxins from a rather narrow assembly of venomous organisms. Besides our recent assessment using *Hexophthalma dolichocephala* ICK-type toxins, only one study on snake venom Kallikrein, one work on *Apis mellifera* Preprosecapin and another work on tailored cell-free systems with tarantula ICK peptides have been carried out [22,23,24]. The exploratory screening provided in this brief report represents the first assessment of cell-free protein production across larger taxonomic and toxinologic scales.

Our work reveals that cell-free protein production in venom biodiscovery is principally attractive as 3Ftx and PLD toxins from snakes and spiders, respectively, were expressed. However, when considering the large diversity of the selected toxins and acknowledging that only four proteins (representing ca. 13% of all chosen compounds) were successfully created, one must conclude that commercially available cell-free systems are probably not very effective for our purpose in their current form. This is also exacerbated when considering the apparent very low protein yield and that previous works have shown that protein folding and disulfide crosslinking are problematic in cell-free animal toxin production [21]. It is therefore to be expected, that even from the herein reported subset of expressed toxins, some may fail to show bioactivity or may be lost completely during purification. However, the fact that we retrieved bands from PLD toxins from *Hemiscorpius* scorpions and especially from *Loxosceles* spiders, a small and cumbersome-to-sample lineage of spiders, suggests that cell-free production harbors some potential. For instance, enzymatic araneomorph spider venom components, such as PLDs, are largely unstudied and often difficult to access. Although in its current state the application of cell-free systems may be limited, we cannot rule out that future modifications and advancements in cell-free technology may soon lead to readily available, commercial systems that may supplement venom biodiscovery. For instance, Wu and colleagues recently carried out work in which they modified an *E. coli*-based cell-free system towards representing an aggregation-free and thermodynamically controlled system (i.e., allowing oxidative folding and refolding of misfolded products) and successfully expressed two ICK peptides [24]. The insights gained by the construction of such novel cell-free systems could be employed to also optimize the formulation of currently marketed products and may pave the way towards their inclusion into venom biodiscovery programs.

## 4. Materials and Methods

### 4.1. Selection of Candidates

**Figure 2:**
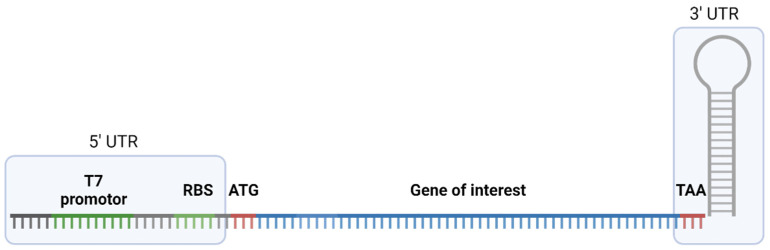
Structure of designed gene fragments comprising a 5’ UTR which includes T7 promotor and a ribosome binding site (RBS), followed by a start codon (ATG), the gene of interest and a 3’UTR site with a stop codon (TAA) and a stem loop based on the T7 terminator sequence. Figure created in Biorender.

In order to explore the suitability of cell-free protein production, we manually selected medically and/or pharmacologically interesting toxins from various animal lineages. Sequences were retrieved from Uniprot [28] or from previously published Venomics works. An overview of selected toxins and all relevant biological and sequence information is given in Supplementary Table S1. We used the NEB PURExpress In Vitro Protein Synthesis System (New England Biolabs, Ipswich, MA, USA) for our assessment. As this is based upon an *E. coli* cell lysate, we optimized the sequences of all toxins towards the *E. coli* codon usage via the EMBOSS package in Geneious 10.0.9. Recombinant DNA fragments encoding the chosen toxins following the previously described general structure were ordered from Invitrogen by Thermo Fisher Scientific (Darmstadt, Germany). The general structure of designed gene fragments is shown in Figure 2.

### 4.2. Cell-Free Production and SDS-PAGE

The NEB PURExpress In Vitro Protein Synthesis System (New England Biolabs, Ipswich, MA, USA) was used to carry out cell-free production. We followed the manufacturer’s recommendations, using 0.2 mL PCR tubes for each reaction. For each reaction, 10% DNA fragment or nuclease-free water (control) were added to the reaction mixture and incubated at 37 °C for 8 h. All tubes were stored at −20 °C after the reaction. Protein synthesis was confirmed by 1D SDS-PAGE. We mixed 1.5 µL of the reaction with 8 µL of 2x Tricine sample buffer containing 2% β-mercaptoethanol and incubated it for 5 min at 95 °C. All samples and references (see Section 4.3) were then loaded onto a 16.5% Mini-PROTEAN Tris-Tricine Gel (Bio-Rad, Hercules, CA, USA) and placed in a Mini-PROTEAN Tetra System chamber (Bio-Rad, Hercules, CA, USA) filled with 1× Tris/Tricine/SDS running buffer. After electrophoresis at 100 V for 100 min, the gel was Coomassie stained with Roti-Blue quick solution (Carl Roth, Karlsruhe, Germany) for 3 h and destained with 10% ethanol in water over night (16 h). We validated the success rate of each toxin by identifying expression bands at the corresponding size for each targeted protein. All reactions were carried out three times and, in order to be considered successful, at least one reaction per triplicate had to show an expression band.

### 4.3. Estimation of Expression Yields

To gain a preliminary insight into the protein yields, a comparison between the intensities of the expression bands and those of reference bands with known protein concentrations was carried out. This method commonly serves as reliable and easy to perform estimation of protein yields. Therefore, we prepared three bovine serum albumin (BSA, G-Biociences, St. Louis, MO, USA) references with concentrations of 66.67 ng/µL, 33.33 ng/µL and 16.67 ng/µL BSA. To achieve BSA concentrations of 200 ng, 100 ng and 50 ng per well for the gel electrophoresis, we mixed 3 µL of each BSA dilution with 8 µL 2×xTricine sample buffer containing 2% β-mercaptoethanol and incubated for 5 min at 95 °C. Together with the samples described in Section 4.2, gel electrophoresis was carried out. The intensities of the BSA reference bands were compared with those of the expression bands and used to calculate an estimation of the yielded protein concentration. 

## Figures and Tables

**Table 1:** Selected toxins for cell-free expression. The table shows toxins selected to explore the potential of cell-free protein production in venom biodiscovery. Given are the toxin name, ID and/or sources, producing organism and its size. Full data of each toxin, including its sequence, are given in Supplementary Table S1.

Toxin Group	Toxin Name	UniProt ID or Source	Source Organism	Lineage	Size [kDa]
Cysteine-rich neurotoxins	U-Asilidin(1)-Mar1a	P0DQI8	*Machimus arthriticus* (Zeller, 1840)	Robber fly	3.1
	U-Asilidin(1)-Eru1a	P0DQJ1	*Eutolmus rufibarbis* (Meigen, 1820)	Robber fly	3.1
	Delta-miturgitoxin-Cp1a	C0HKG7	*Cheiracanthium punctorium* (Villers, 1789)	Spider	15.1
	Omega Hexatoxin Hi1a	P0C2L5	*Hadronyche infensa* (Hickman, 1964)	Spider	3.9
	Omega-theraphotoxin-Cc1a	D5J6X1	*Pelinobius muticus* (Karsch, 1885)	Spider	4.3
	U11-pisautoxin-Dm1a	S5MK94	*Dolomedes mizhoanus* (Kishida, 1936)	Spider	6.8
	U18-barytoxin-Tl1a	W4VRU3	*Trittame loki* (Raven, 1990)	Spider	7.5
	U1-barytoxin-Tl1a	W4VRV2	*Trittame loki* (Raven, 1990)	Spider	11.1
	U1-oxotoxin-Ot1a	W0LQ84	*Oxyopes takobius* (Andreeva & Tyschchenko, 1969)	Spider	13.7
	U1-pisautoxin-Dm1a	S5N3Q8	*Dolomedes mizhoanus* (Kishida, 1936)	Spider	11.4
	U1-TRTX-Lp1b	P61506	*Lasiodora parahybana* (Mello-Leitao, 1917)	Spider	5.7
Protease inhibitor	Hirudin	P28509	*Hirudo medicinalis* (Linnaeus, 1758)	Leech	7.0
Antimicrobial peptide	Lycotoxin	B6DD06	*Lycosa singoriensis* (Laxmann, 1770)	Spider	4.8
(Putative) Enzyme	Phospholipase D	A0A1L4BJ98	*Hemiscorpius lepturus* (Peters, 1861)	Scorpion	33.0
	Phospholipase D	Q8I914	*Loxosceles laeta* (Nicolet, 1849)	Spider	32.1
	Phospholipase D	Q1KY80	*Loxosceles laeta* (Nicolet, 1849)	Spider	32.0
	Phospholipase D	Q1KY79	*Loxosceles laeta* (Nicolet, 1849)	Spider	32.6
	Phospholipase D	C0JB23	*Loxosceles laeta* (Nicolet, 1849)	Spider	31.5
	Phospholipase D	Q8I912	*Loxosceles laeta* (Nicolet, 1849)	Spider	31.7
	CAP	[25]	*Argiope bruennichi* (Scopoli, 1772)	Spider	45.3
Three-finger toxin (3Ftx)	Long neurotoxin 1	P34074	*Naja annulata* (Peters, 1876)	Snake	7.8
	Delta-elapitoxin-Cb1a	P0DL82	*Calliophis bivirgatus* (Boie, 1827)	Snake	6.7
	Scutelatoxin	Q4VRI0	*Oxyuranus s. scutellatus* (Peters, 1876)	Snake	6.6
	Cytotoxin 1	P01455	*Naja annulifera* (Peters, 1854)	Snake	6.7
	Cytotoxin sagitoxin	P83345	*Naja sagittifera* (Wall, 1913)	Snake	6.8
Others	Pimplin	Q8WPC8	*Pimpla hypochondriaca* (Retzius, 1783)	Wasp	13.1
	Ryncolin-1	D8VNS7	*Cerberus rynchops* (Schneider, 1799)	Snake	36.4
	Ryncolin-2	D8VNS8	*Cerberus rynchops* (Schneider, 1799)	Snake	36.6
	Ryncolin-3	D8VNS9	*Cerberus rynchops* (Schneider, 1799)	Snake	36.4
	Ryncolin-4	D8VNT0	*Cerberus rynchops* (Schneider, 1799)	Snake	36.2

## Data Availability

The data presented in this study are available in the manuscript and in the supplementary Material.

## Supplementary Materials

The following supporting information can be downloaded at: https://www.mdpi.com/article/10.3390/ijms25158286/s1.
